# On the information hidden in a classifier distribution

**DOI:** 10.1038/s41598-020-79548-9

**Published:** 2021-01-13

**Authors:** Farrokh Habibzadeh, Parham Habibzadeh, Mahboobeh Yadollahie, Hooman Roozbehi

**Affiliations:** 1 R&D Headquarters, Petroleum Industry Health Organization Polyclinic, Eram Blvd, 7143837877 Shiraz, Iran; 2 Persian BayanGene Research and Training Center, Shiraz University of Medical Sciences, Shiraz, Iran; 3 Peyvand Clinical Laboratory, Shiraz, Iran

**Keywords:** Classification and taxonomy, Data mining, Statistical methods, Biomarkers, Medical research, Prostate

## Abstract

Classification tasks are a common challenge to every field of science. To correctly interpret the results provided by a classifier, we need to know the performance indices of the classifier including its sensitivity, specificity, the most appropriate cut-off value (for continuous classifiers), etc. Typically, several studies should be conducted to find all these indices. Herein, we show that they already exist, hidden in the distribution of the variable used to classify, and can readily be harvested. An educated guess about the distribution of the variable used to classify in each class would help us to decompose the frequency distribution of the variable in population into its components—the probability density function of the variable in each class. Based on the harvested parameters, we can then calculate the performance indices of the classifier. As a case study, we applied the technique to the relative frequency distribution of prostate-specific antigen, a biomarker commonly used in medicine for the diagnosis of prostate cancer. We used nonlinear curve fitting to decompose the variable relative frequency distribution into the probability density functions of the non-diseased and diseased people. The functions were then used to determine the performance indices of the classifier. Sensitivity, specificity, the most appropriate cut-off value, and likelihood ratios were calculated. The reference range of the biomarker and the prevalence of prostate cancer for various age groups were also calculated. The indices obtained were in good agreement with the values reported in previous studies. All these were done without being aware of the real health status of the individuals studied. The method is even applicable for conditions with no definite definitions (e.g., hypertension). We believe the method has a wide range of applications in many scientific fields.

## Introduction

Classification tasks are a common challenge to every field of science. We often need to categorize a new observation into one of predefined groups based on its attributes. The definitions provided for a classifier varies a little bit from field to field. In statistics, a classifier is an algorithm that help us with data categorization^1^. In machine learning, the function of a classifier is to map objects based on their features to classes^2^. In medicine, a classifier can be considered a diagnostic test helping physicians to classify people to healthy and diseased groups^3^. Classifiers are extensively used in various disciplines of science. A classifier can be used for categorization of an incoming e-mail into spam and non-spam, based on its contents^4^; stars into spectral types, based on their diffraction pattern^5^; and tumors to benign or malignant, based on a biomarker^6^, to name just a few applications.

There are several performance indices for the assessment of a classifier. The performance of a binary classifier in classifying a binary attribute, typically involves comparing results of two methods—a gold-standard method and the classifier being tested. In this way, we can determine the true-positive rate or sensitivity (*Se*), the true-negative rate or specificity (*Sp*), and the likelihood ratio of a positive or negative results for the classifier^7^. Typically, several studies should be conducted to find these values. Herein, we show that all these indices are already available, hidden in the distribution of the variable used to classify and can readily be harvested. We present a method to harvest the indices. An educated guess about the distribution of the variable used to classify in each class would help us to decompose the frequency distribution of the variable in population into its components—the probability density function of the variable in each class. Based on the harvested parameters, we can then calculate the performance indices of the classifier. As an example for the application of the proposed method, we use the frequency distribution of prostate-specific antigen (PSA), an immunologic biomarker commonly used for the diagnosis of prostate cancer, and compare the results to values reported in previous studies.

### Case study

PSA is commonly used for the diagnosis of prostate cancer. In this case study, we use PSA values for the categorization of patients into two groups of “non-diseased” and “diseased.” However, to correctly interpret the PSA results, we need to know the reference range of the test value; the most appropriate cut-off value for PSA above which the test is considered positive^3^; what percentage of a population has the disease (prevalence or the prior probability of the disease [*pr*]); the probability that the test becomes positive in a diseased person (true-positive rate or test sensitivity [*Se*]); the probability that a non-diseased person becomes test-negative (true-negative rate or test specificity [*Sp*]); the false-positive and false-negative rates and an estimation of their costs; the likelihood ratio of a positive or negative test result or the likelihood ratio for a certain PSA value^6,8^, etc. Several studies have so far been conducted to find all these indices. Herein, we harvest these values from the frequency distribution of PSA measured in a group of people. Nonetheless, the very first step is taking an educated guess about the distribution of PSA in non-diseased and diseased people.

#### Hypothesis about the distribution of PSA

The distribution of PSA in healthy people and those with prostate cancer is positively skewed. However, with acceptable clinical accuracy, after a logarithmic transformation, it has a normal distribution in both non-diseased and diseased people; Ln(PSA) has a binormal distribution in the population (consisting of both non-diseased and diseased individuals)^9^.

The general form of a normal distribution with a mean of *µ* and the standard deviation (SD) of *σ* is^3^:

1 $$\begin{gathered} f\left( {x|\mu ,\sigma^{2} } \right) = \frac{1}{{\sqrt {2\pi } \,\sigma }}e^{{\frac{{ - \left( {x - \mu } \right)^{2} }}{{2\sigma^{2} }}}} \\ = \frac{1}{\sigma }\varphi \left( {\frac{x - \mu }{\sigma }} \right) \\ \end{gathered}$$

where *φ* represents the probability density function of the distribution. A binormal distribution, the superposition of two normal distributions, thus, has the following general equation:

2 $$y(x) = \frac{{a\left( {1 - pr} \right)}}{{\sigma_{1} }}\varphi \left( {\frac{{x - \mu_{1} }}{{\sigma_{1} }}} \right) + \frac{a\,pr}{{\sigma_{2} }}\varphi \left( {\frac{{x - \mu_{2} }}{{\sigma_{2} }}} \right)$$

where, *μ*_1_*, σ*_1_*, μ*_2_*,* and *σ*_2_ represent mean and the SD of Ln(PSA) in the non-diseased and diseased people, respectively; *pr* represents the prior probability (prevalence, if no other information is available) of the disease; and *a* is a correction factor.

Equation 2 is a little bit different from the three-parameter binormal equation commonly mentioned in the literature^10^. The three-parameter distribution, in fact, includes two half-Gaussian distributions. Each point of the distribution in Eq. 2 is the summation of two Gaussian distributions. Choosing Eq. 2 for our model was rooted in our hypothesis that normal prostate cells are a homogenous population of cells producing PSA, the amount of which increases by age^11^. Prostate malignant cells, on the other hand, are heterogenous in genotype and produce a wide range of PSA, the amount of which is not age-dependent^12,13^. We therefore assumed two independent populations of cells—a population of homogenous normal prostate cells producing PSA (the first term in Eq. 2) with an age-dependent distribution, and a population of heterogenous prostate malignant cells producing PSA (the second term in Eq. 2) the distribution of which is not age-dependent.

#### Frequency distribution of PSA

A large set of data, consisting of 18,561 records of PSA values taken from the database of a general clinical laboratory in Shiraz, southern Iran, were used for analysis. PSA values were measured between March 2017 and September 2019. The values were then log-transformed. A histogram of the relative frequency distribution of Ln(PSA), was then constructed.

## Results

An educated guess would help us to decompose the frequency distribution of the variable used to classify in the population to its components—the probability distribution function of the variable in each group. Using a nonlinear curve fitting, a binormal curve (our hypothesized distribution) was fitted to the relative frequency distribution of Ln(PSA); the parameters of the probability density functions of the variable for non-diseased and diseased groups were derived. Based on the harvested parameters, the classifier performance indices were then calculated.

### Frequency distribution of PSA

**Figure 1:**
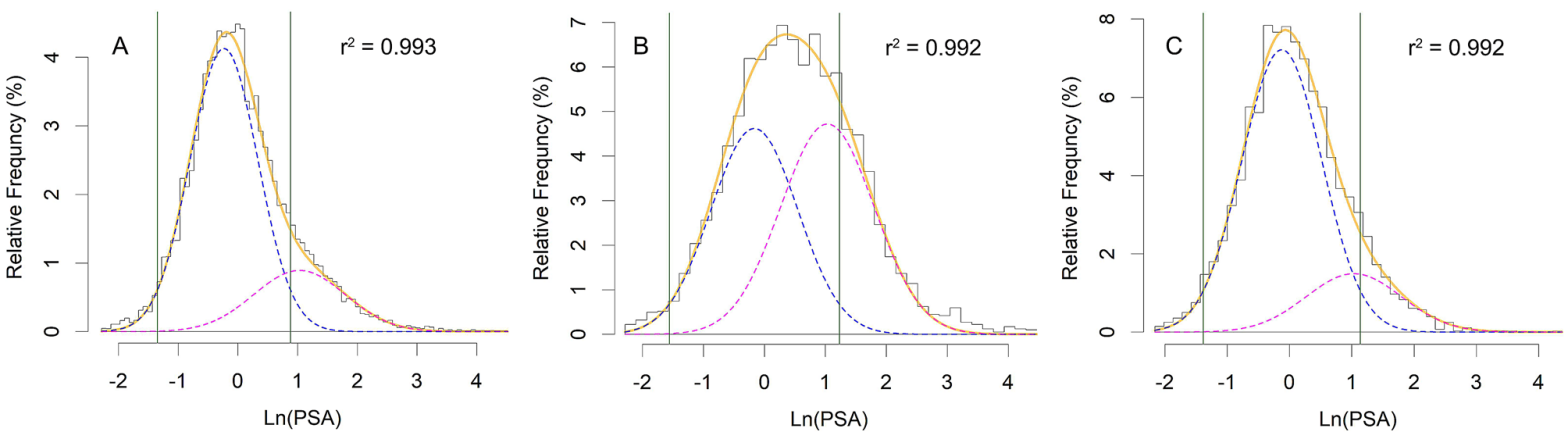
Histograms of the relative frequency distribution of Ln(PSA) for (A) those aged ≥ 20 years, (B) ≥ 65, and (C) 54–59 years. In each panel, the orange solid curve is the binormal curve fitted to the data (the histogram representing the relative frequency). The curve is in fact the result of superposition of two normal curves describing the relative frequency distribution of Ln(PSA) in non-diseased individuals (blue dashed curve) and diseased patients (magenta dashed curve). Vertical green solid lines bound the reference range for PSA.

The relative frequency of Ln(PSA), as expected, had a good (r^2^ = 0.993) fit to the hypothsized binormal distribution (Fig. 1A). Analysis of the subset data revealed that the distribution of those aged ≥ 65 years (n = 4024) also had a good fit (r^2^ = 0.992, Fig. 1B). In this subset, the maximum value for the distribution of the diseased people (Fig. 1B, magenta dashed curve) was almost equal to that of non-diseased people (blue dashed curve), indicating that the prevalence of the disease, *pr* in Eq. 2, is around 50%. Having a similar weight, under this situation, *μ*_2_ and *σ*_2_ would be calculated with the same accuracy as *μ*_1_ and *σ*_1_ are.

Considering our hypothesis that there are two cell populations (non-diseased and diseased), and that the distribution of PSA in the diseased group is not age-dependent, we used the estimations for *μ*_2_ and *σ*_2_ (the mean and SD of Ln(PSA) for diseased population) that came from this age group for all other analyses. Assuming these values, the relative frequency distribution of Ln(PSA) for those aged 54–59 years (n = 2781) had also a good fit (r^2^ = 0.992, Fig. 1C). In fact, this assumption resulted in an acceptable fit (r^2^ > 0.965) of a binormal distribution (Eq. 2) to the observed distribution of data for all age groups studied.

### Reference range of PSA

**Figure 2:**
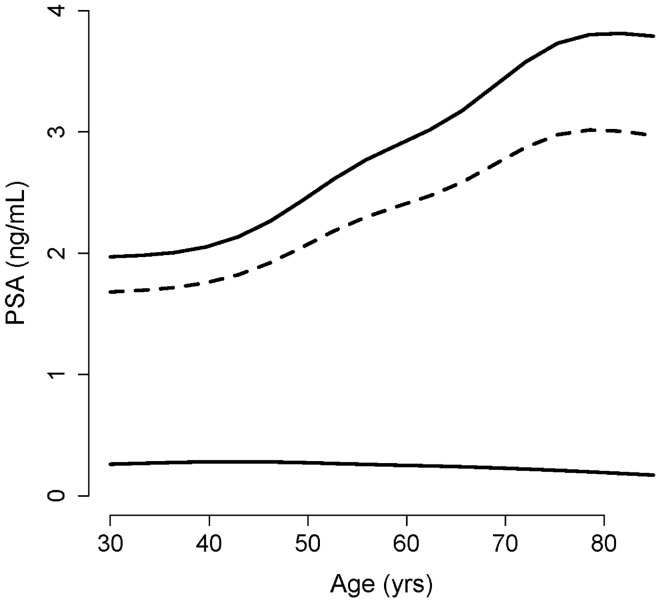
The reference range of the disease for each age. The values were obtained from subset analysis of data of men aged 20–39, 40–49, 50–59, 60–69, 70–79, and ≥ 80 years. The two solid lines represent the lower and upper limits of the reference range; dashed line, the 95th percentile of PSA. The values for each age group were calculated based on the mean and SD of the distribution of Ln(PSA) in non-diseased people in each subset^9^.

The reference range for a diagnostic test, here PSA, is important for clinicians. The range is commonly defined as the interval between the 2.5th and 97.5th percentiles of the test value distribution in an apparently healthy population^14^. Presuming a normal distribution for Ln(PSA) in non-diseased individuals and given the mean and SD of the distribution (*μ*_1_ and *σ*_1_ for the dashed blue curves in Fig. 1) for each age group, we calculated the reference range (*μ*_1_ ± 1.96 *σ*_1_) for Ln(PSA) (Fig. 1, the region bounded by vertical green solid lines), and thus for PSA, as well as the 95th percentile of PSA for each age group (Fig. 2). The width of the interval increased with age. This was mainly attributed to the increasing upper limit of the reference range with age, an observation in concordance with what has been reported earlier in several studies conducted on large number of people of various ethnic backgrounds^15–20^.

### Prevalence of the disease

**Figure 3:**
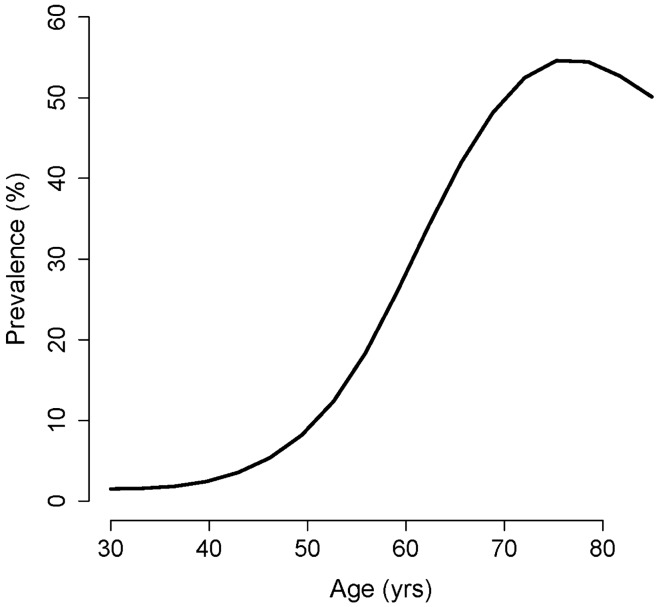
The prevalence of the disease for each age. The values were obtained from subset analysis of data of men aged 20–39, 40–49, 50–59, 60–69, 70–79, and ≥ 80 years. The prevalence came directly from the curve fitting.

The prevalence of the disease for each age group was easily derived from the curve fitting procedure. For example, the prevalence of the disease for 54–59-year-old men (n = 2781) was 19.8%. There was a sharp increase in the prevalence between the age of 45 and 75 years (Fig. 3). It decreased thereafter. Our results were in line with the reported results in the literature. Even, the observation that the prevalence decreased slightly after the age of 75 was in line with the reported decrease in the incidence of the prostate cancer after this age^21,22^.

There is a significant variation in the reported prevalence of prostate cancer among different populations. A systematic review of published autopsy studies shows that the prevalence varies nonlinearly from 5% in those aged < 30 years to 59% in men aged > 79 years^23^. Another forensic autopsy study conducted in Japan shows that the prevalence increases by age—10.9% in 50’s, 17.1% in 60’s, and 21.2% in 70’s^24^. The corresponding values we calculated were 16.5%, 40.6%, and 54.5%, respectively (Fig. 3). The observed difference mainly originated from our definition of the disease. While autopsy studies use various techniques (histopathology, histochemical staining, etc.) to identify malignant cells in tissue specimens, our method defines the disease based on the distribution of PSA the prostate cells produce.

Tumors are generally not clinically detectable until they become as large as 1 cm in diameter (≈ 10^9^ cells)^12^. This represents around 75% of the natural history of the tumor; we can only diagnose a fraction of the tumors; and that is why the prevalence of tumors in autopsy studies is much higher than that reported in clinical settings. Nevertheless, there are tumors that are too small to be even identified in autopsy, whereas our method, which is based on the distribution of PSA, can theoretically predict their presence; our method is even more sensitive than the current autopsy techniques and that is why the prevalence we found was higher than the reported figures in autopsy studies. The difference was higher for older men because the chance of developing malignant cells significantly increased with age^22^.

Prostate cancer is typically a slow progressive disease. It has a long latent phase and many men with prostate cancer die from other causes before their cancer being diagnosed at all^24^. This is why the reported prevalence in live people diagnosed in a clinical setting is much lower than those reported in autopsy studies. For example, in a study conducted in Japan, 170 of almost 24,000 men in their 60’s screened for prostate cancer, were found positive, which translates to a prevalence of only 0.7%^11^. The study reports a prevalence of 0.3% in those aged 54–59; we came to a value of 19.8%. The tumor size in many of these people is too small to cause any problems during the rest of their lives. These small tumors cannot be identified using the currently available diagnostic modalities. The observed difference might also arise from the difference in the distribution of PSA among races^19^.

Based on our model, 67.8% of diseased people and 82% of 62–91-year-old men had a PSA ≤ 4 ng/mL. The prevalence of the disease among this age group was 49.4%. Based on Bayes’ theorem^8,25^, the probability that a man aged 62–91 years with a PSA ≤ 4 ng/mL had cancer was 40.8%:

$$\begin{gathered} P(D^{ + } |PSA \le 4) = \frac{{P(PSA \le 4|D^{ + } )}}{P(PSA \le 4)}P(D^{ + } ) \\ = \frac{0.678}{{0.820}} \times 0.494 = 0.408 \\ \end{gathered}$$

where *D*^+^ represents presence of disease, and *P*(*A* | *B*) is the conditional probability of event *A* happens given the event *B* has happened^25^. Considering the limitation of our current diagnostic tools mentioned above, this value is in good agreement with the previous reported figure of 0.152 in the United States^26^.

### Calculation of the sensitivity, specificity, the receiver operating characteristic (ROC) curve, and the cut-off value

**Figure 4:**
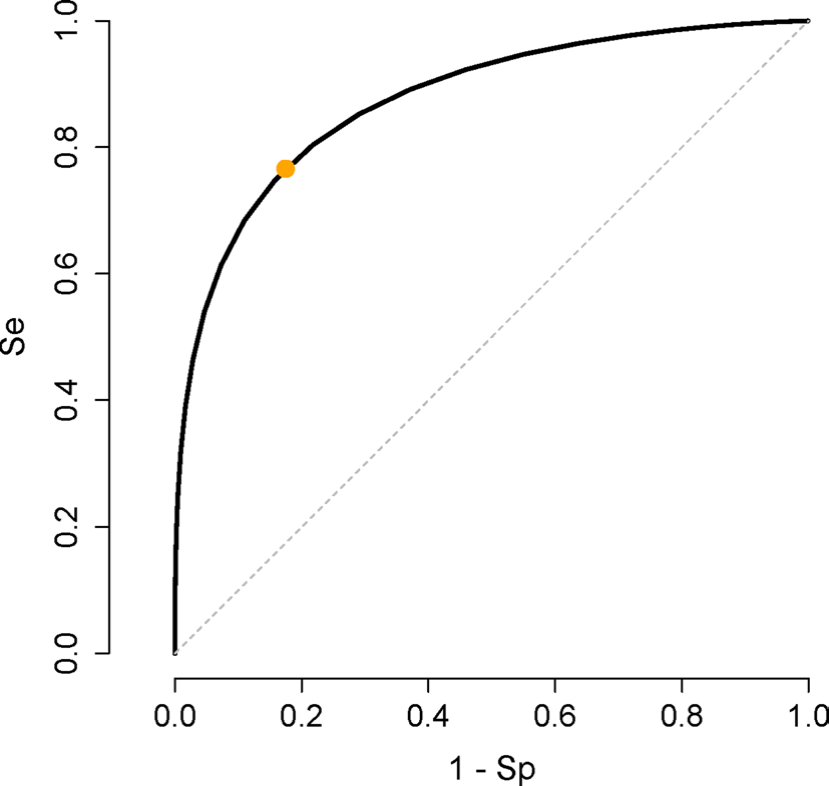
ROC curve for 54–59-year-old men. The curve is constructed based on subset analysis of 54–59-year-old men. The area under the curve is 0.874. The orange solid circle corresponds to the most appropriate cut-off value for PSA, 1.61 ng/mL, a point maximizing the Youden’s index (corresponding to a relative cost of a false-negative to a false-positive test result of 4), corresponding to a *Se* and *Sp* of 76.6% and 82.5%, respectively.

Given the means and SDs of Ln(PSA) in diseased and non-diseased people and considering normal distribution for both diseased and non-diseased people (based on our hypothesis), using Eq. 12, we calculated the *Se* and *Sp* of the test assuming a cut-off value of *t* for Ln(PSA) as follows^3^:

3 $$\begin{gathered} Se(t) = \frac{1}{{\sigma_{2} }}\int\limits_{t}^{ + \infty } {\varphi \left( {\frac{{x - \mu_{2} }}{{\sigma_{2} }}} \right)\,dx} \\ = 1 - \Phi \left( {\frac{{t - \mu_{2} }}{{\sigma_{2} }}} \right)\, \\ \end{gathered}$$

and

4 $$\begin{gathered} Sp(t) = \frac{1}{{\sigma_{1} }}\int\limits_{ - \infty }^{t} {\varphi \left( {\frac{{x - \mu_{1} }}{{\sigma_{1} }}} \right)\,dx} \\ = \Phi \left( {\frac{{t - \mu_{1} }}{{\sigma_{1} }}} \right)\, \\ \end{gathered}$$

where Φ represents the cumulative distribution function of the standard normal distribution. These calculations were done for each age group. For example, for 54–59-year age group, *μ*_1_*, σ*_1_*, μ*_2_*,* and *σ*_2_ were -0.124, 0.643, 1.033, and 0.766, respectively. Using Eqs. 3 and 4, the *Se* and *Sp* for a series of Ln(PSA) cut-off values were calculated, based on which an ROC curve was constructed (Fig. 4)^27,28^. The area under the ROC curve was 0.874.

Based on the prevalence (19.8% for 54–59-year-old men in our example), and the cost of a false-negative relative to a false-positive test result (arbitrarily chosen to be 4 in our example), the most appropriate test cut-off value was calculated to be 1.61 ng/mL (the orange solid circle, Fig. 4). It corresponds to a *Se* and *Sp* of 76.6% and 82.5%, respectively^3^. This cut-off value also maximized the Youden’s index (*Se* + *Sp* − 1), an index reflecting maximum potential effectiveness of a biomarker^3,29^.

The most appropriate test cut-off value could be calculated for each age group. However, the cost of a false-negative test result relative to a false-positive test result needs to be considered separately for different age groups. For example, one may estimate it at 4 for 54–59-year-old men, and 0.2 for those aged 80 or more, as early diagnosis of the disease is not always good; it would increase the likelihood of harm rather than benefit because of detection of clinically insignificant diseases, particularly at old ages^30^. Furthermore, the clinical relevance of the inconclusive results obtained should be considered. There are various ways to handle the inconclusive results. Two graphs ROCs is the easiest strategy to be used^31^; other techniques are more complex involving mathematical rigors^32,33^.

A study conducted in Japan proposed a cut-off value of 2.3 ng/mL for PSA in those aged 54–59 years^11^. This value is less than the cut-off value of 3.0 set by the Japanese Urological Association for the age group. The cut-off value of 2.3 ng/mL is associated with a test *Se* of 100% and a *Sp* of 92.3%^11^. Based on our results, however, the most appropriate cut-off value was 1.61 ng/mL, which was associated with a *Se* and *Sp* of 76.6% and 82.5%, respectively. The observed difference originated again from our case definition. While our model considers those with just a few malignant cells “diseased,” the aforementioned study failed to diagnose the disease in them and thus falsely categorized them into “non-diseased” group. This is also why in our model we set the cut-off to a lower value, which decreases the test *Sp*. If we would have set our cut-off to 2.3 ng/mL (and missed many diseased people), the *Sp* became 90.3%, very well comparable with that reported in the study^11^.

While some conditions, like prostate cancer, do have well-defined clinicopathological definitions, certain disease conditions do not. For example, hypertension does not have such a well-defined clinicopathological definition. What we know for sure is that a higher blood pressure is associated with higher mortality and morbidity. Nonetheless, we cannot define hypertension or give a certain cut-off value for classifying people according to their blood pressure and assess the risk they bear. The cut-off values proposed for the diagnosis of hypertension are constantly changing over time^34^. Our proposed method can probably identify the group of hypertensive and non-hypertensive people, and provide the reference range for blood pressure and the prevalence of hypertension for each age group. In this way, we can have, once and for all, an invariant definition for hypertension.

### Positive and negative predictive values

For a given cut-off value of *t*, and the prevalence of the disease, we calculated the positive (*PPV*) and negative predictive values (*NPV*) using the following equations:

5 $$\begin{gathered} PPV(t) = \frac{pr\,Se(t)}{{pr\,Se(t) + \left( {1 - pr} \right)\left( {1 - Sp(t)} \right)}} \hfill \\ NPV(t) = \,\frac{{\left( {1 - pr} \right)Sp(t)}}{{\left( {1 - pr} \right)Sp(t) + pr\left( {1 - Se(t)} \right)}} \hfill \\ \end{gathered}$$

Given the *Se*, *Sp*, *pr* of the disease, and the cut-off value of 1.61 ng/mL for PSA calculated for those aged 54–59 years, the *PPV* and *NPV* were 51.9% and 93.5%, respectively (Eq. 5).

### Likelihood ratios

Positive and negative likelihood ratios (*LR*^+^ and *LR*^*–*^, respectively) for a given cut-off value of *t*, were calculated as follows^8^:

6 $$\begin{gathered} LR^{ + } (t) = \frac{Se(t)}{{1 - Sp(t)}} \hfill \\ LR^{ - } (t) = \,\frac{1 - Se(t)}{{Sp(t)}} \hfill \\ \end{gathered}$$

*LR*^+^ and *LR*^*–*^ for the cut-off value of 1.61 ng/mL were 4.38 and 0.28, respectively (Eq. 6).

Having *Se* and *Sp* for two PSA values, it is possible to calculate the likelihood ratio for a range of PSA^8^. For example, based on the information obtained, the *Se* and *Sp* for a cut-off value for Ln(PSA) of 1.79 (PSA of 6 ng/mL) were 16.1% and 99.9%, respectively; they were 32.2% and 99.1%, respectively, for a cut-off value for Ln(PSA) of 1.39 (PSA of 4 ng/mL). The likelihood ratio for having a PSA between 4 and 6 ng/mL is thus^8^:

7 $$LR(t_{1} \le PSA < t_{2} ) = - \frac{{Se(t_{1} ) - Se(t_{2} )}}{{Sp(t_{1} ) - Sp(t_{2} )}}$$

Therefore,

$$LR(4 \le PSA < 6) = - \frac{0.161 - 0.322}{{0.999 - 0.991}} = 20$$

meaning that the probability of observing a PSA level between 4 and 6 ng/mL is 20 times more often in 54–59-year-old men with the disease compared with those without the disease^8^.

**Figure 5:**
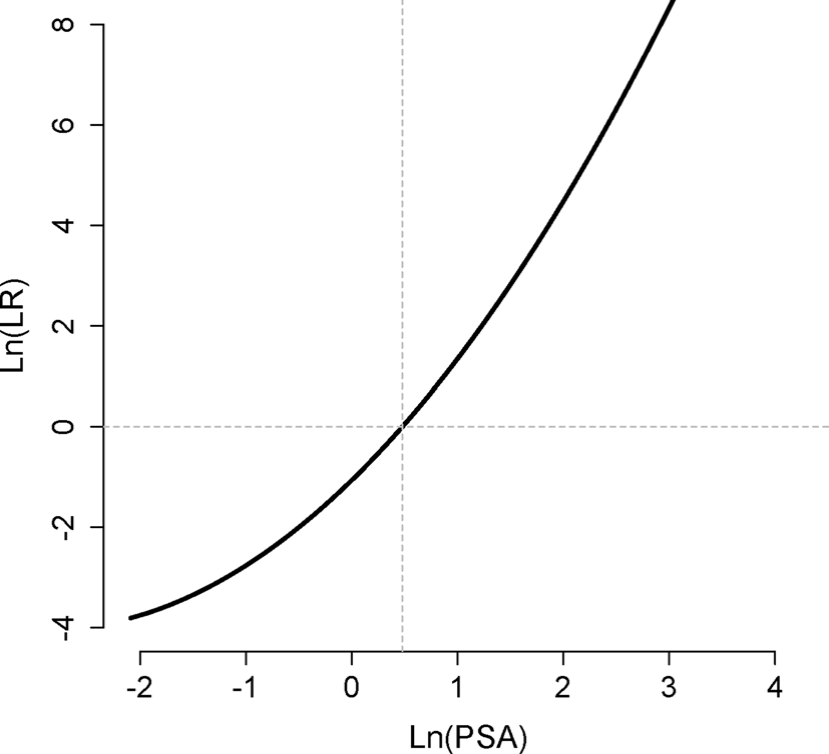
Likelihood ratio (*LR*) for various PSA values in 54–59-year-old men. Note that both axes are logarithms of the variables. The horizontal gray dashed line corresponds to an *LR* of 1; the vertical gray dashed line represents the cut-off value, PSA of 1.61 ng/mL.

Furthermore, because we derived the probability density function for the non-diseased and diseased groups, we could calculate the likelihood ratio (*LR*) for any given PSA values in the studied group (e.g., 54–59-year-old men) using the specific form of Eq. 13 (Fig. 5)^8^:

8 $$LR(x) = \frac{{\sigma_{1} \,\varphi \left( {\frac{{x - \mu_{2} }}{{\sigma_{2} }}} \right)}}{{\sigma_{2} \,\varphi \left( {\frac{{x - \mu_{1} }}{{\sigma_{1} }}} \right)}}$$

where *x* is Ln(PSA). This is technically the slope of the line tangent to the ROC curve at the point corresponding to the PSA value. In practice, it is very difficult to calculate this slope accurately, as the ROC curve is in fact just a set of discrete points, not a differentiable curve^8^. Our method, however, can readily provide the information.

For example, the *LR* for Ln(PSA) of 1.79 (PSA of 6 ng/mL) was 43, meaning that in the age group of 54–59 years, a PSA level of 6 ng/mL was 43 times more likely to be observed in those with the disease as compared with those without the disease^8^. On the other hand, the *LR* for Ln(PSA) of 0 (PSA of 1 ng/mL) was 0.34; the likelihood of observing a PSA of 1 ng/mL in diseased people was one-third of those without the disease.

## Conclusion

We showed that if we have an educated guess about the distribution of the variable used to classify, we can harvest the reference range for the variable; the prior probability of the condition of interest (here, prevalence of a disease); the classifier performance indices including its *Se, Sp, PPV, NPV, LRs*, also the ROC curve and the area under the curve, and the most appropriate cut-off value. However, to be sure about the frequency distribution of the variable we need to perform an extensive sampling over all possible range of its values. Particular attention should be paid to the extremes (tails) that might differentiate between the two classes, healthy and non-healthy individuals.

In our case study, we assumed a binormal distribution for Ln(PSA) for a well-defined dichotomous condition, diseased and non-diseased people. The method can be applied as well to those conditions without a well-defined clinicopathological definition, say hypertension, and to other scientific disciplines.

The results calculated in our case study were based on the relative frequency distribution of the PSA and an educated guess about the distribution of PSA in non-diseased and diseased people. No information about the prostate cancer status of each individual studied was available. Our assumption to take the mean and SD of Ln(PSA) of patients aged ≥ 65 years as an acceptable estimate for *μ*_2_ and *σ*_2_ for all other analyses, would seem unacceptable at first glance, as those aged ≥ 65 made 22% of the total population studied. But, when we take into account that more than half of all prostate cancers reported happen in this age group, the assumption made might look reasonable. Furthermore, we hypothesized that the PSA distribution in diseased people is not age-dependent. As a matter of fact, the essence of the work here is neither choosing the binormal distribution we used for curve fitting nor our choice for *μ*_2_ and *σ*_2_; it is the way we could derive the test indices (*Se, Sp, LRs,* etc.) solely based on an educated guess about the distribution of the variable used to classify, no matter how we become aware of the distribution. In many instances, such as for the PSA^9,31,33^, there are enough evidence to help us with making an educated guess and choosing a few appropriate distributions. Note that, if we had the distributions of the variable for the groups, even in numeric forms, we could have calculated all the aforementioned indices with numerical methods. Knowledge about the distribution of the variable in the classes help us with correct decomposition of the population (non-diseased and diseased) relative frequency distribution (the histogram in Fig. 1) into its components—the relative frequency distribution (and thus the probability density functions) of the variable used to classify in each group (the blue and magenta dashed lines in Fig. 1).

The very good agreement between the results obtained from this method with those obtained independently from prior research studies as well as the field-independency of the method proposed may in some way imply the deep-seated mathematical rules in action in the nature from the microcosm within us and our surrounding world to the macrocosm, the universe. We believe the method has a wide range of applications in many scientific fields.

## Methods

Suppose a continuous classifier with a binary outcome. Let *x* be a random variable used to classify with probability density functions of *f*_*i*_(*x*) for the *i*th class. Let the classes be designated “negative” and “positive.” Assume the lower values of the classifier belong to the negative class; the higher, to the positive. Comparing the results of the classification against a gold-standard method gives four outcomes—true-positive (*TP*), false-positive (*FP*), true-negative (*TN*), and false-negative (*FN*).

### Prior probabilities

Assume that *x* is the variable used for the classification, and that *pr*_*i*_ designates the probability that a randomly selected value of *x* being classified to class *i*—the prior probability. Then, the frequency distribution for the variable *x* observed in the population, regardless of classes, should follow the general equation:

9 $$F(x) = A\,\sum\limits_{i = 1}^{n} {pr_{i} \,f_{i} (x)}$$

where *A* is a constant and 0 ≤ *pr*_*i*_ ≤ 1 for all *i*’s, and

10 $$\,\sum\limits_{i = 1}^{n} {pr_{i} = 1}$$

An educated guess about the general form of the distribution of the variable used to classify in each class would help us to decompose the frequency distribution of the variable in population, *F*(*x*), into its components—the probability density function of the variable in each class, *f*_*i*_(*x*). We may estimate the *pr*_*i*_ and the parameters of the distributions with nonlinear curve fitting procedures. Based on the harvested parameters, we can then calculate the performance indices of the classifier.

### Performance indices of the classifier

**Figure 6:**
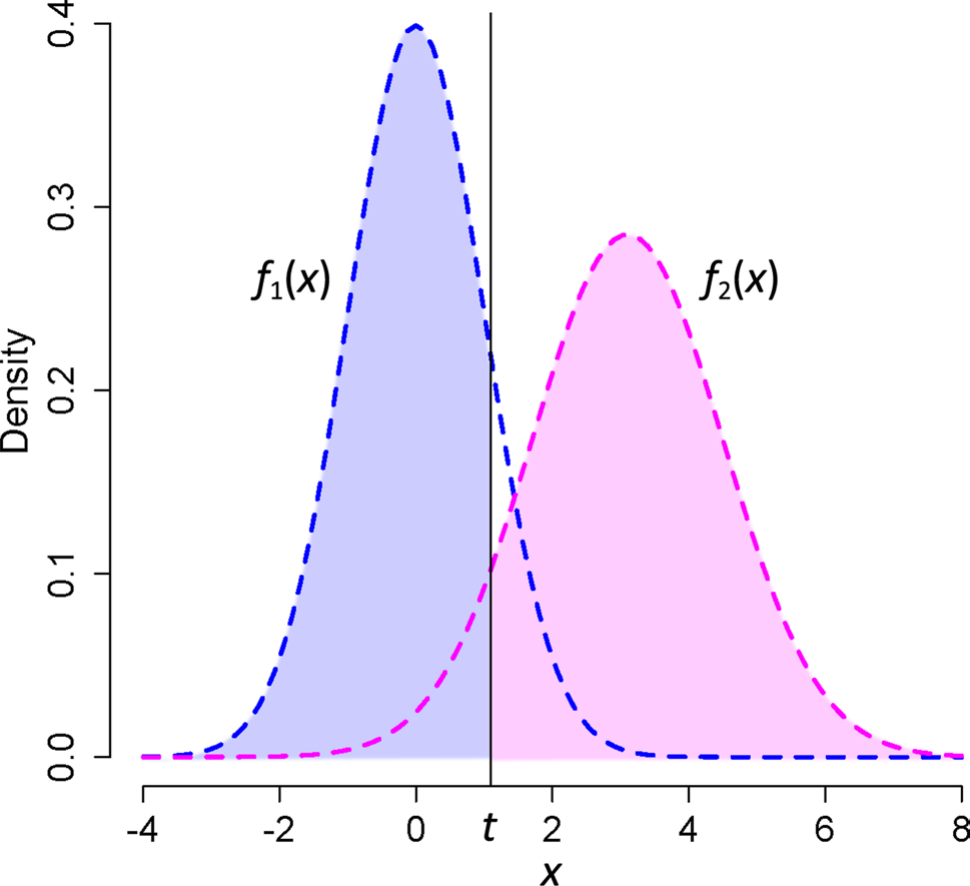
An example of the probability density functions of a continuous variable *x* used to classify the input value to binary outcome (say a diagnostic test) for the negative class (*f*_1_(*x*), blue dashed line) and positive class (*f*_2_(*x*), magenta dashed line). The negative and positive classes may correspond to non-diseased and diseased people, respectively. The cut-off value of *t* is represented by the vertical solid line. The area under *f*_2_(*x*) to the right of the cut-off value (the pink region) is then the sensitivity (*Se*), and the area under *f*_1_(*x*) to the left of the cut-off value (the light blue region) is the specificity (*Sp*).

For a binary classifier (Fig. 6), the sensitivity (*Se*) and specificity (*Sp*) of the classifier are defined as:

11 $$\begin{gathered} Se = \frac{TP}{{TP + FN}} \\ Sp = \frac{TN}{{TN + FP}} \\ \end{gathered}$$

The *Se* and *Sp* are functions of the classifier used. As an example, for our binary classifier (Fig. 6), for a cut-off value of *t*, the functions are^3^:

12 $$\begin{gathered} Se(t) = \int\limits_{t}^{ + \infty } {f_{2} (x)\,dx} \\ Sp(t) = \int\limits_{ - \infty }^{t} {f_{1} (x)\,dx} \\ \end{gathered}$$

### The receiver operating characteristic curve and the cut-off value

Having *Se* and *Sp* corresponding to various values of the variable used to classify, we can draw the receiver operating characteristic (ROC) curve of the classifier and determine its most appropriate cut-off value^3^.

### Likelihood ratio

For a binary classifier, the positive and negative likelihood ratio (*LR*^+^ and *LR*^–^, respectively) associated with a cut-off value of *t*; the likelihood ratio for an interval [*t*_1_, *t*_2_), *LR*(*t*_1_ ≤ *x* < *t*_2_); and the likelihood ratio for a certain value *x*, *LR*(*x*); can be derived as follows^8^:

13 $$\begin{gathered} LR^{ + } (t) = \frac{Se(t)}{{1 - Sp(t)}} \hfill \\ LR^{ - } (t) = \,\frac{1 - Se(t)}{{Sp(t)}} \hfill \\ LR(t_{1} \le x < t_{2} ) = - \frac{{Se(t_{1} ) - Se(t_{2} )}}{{Sp(t_{1} ) - Sp(t_{2} )}} \hfill \\ LR(x) = \frac{{f_{2} (x)}}{{f_{1} (x)}} \hfill \\ \end{gathered}$$

### Case study

#### Source of data

We analyzed the PSA values taken from the database of a general clinical laboratory in Shiraz, southern Iran. Each day, the laboratory performs between 6000 and 8000 tests on samples received from 600 to 800 people with various health states coming from various parts of Fars province. The values were measured in samples received between March 2017 and September 2019 using electrochemiluminescence immunoassay. To eliminate outliers, we only included the samples of men aged 20 years or above having PSA values between 0.1 and 100 ng/mL.

#### Data analysis

Data analysis was performed with *R* software ver 4.0.2 (2020-06-22).

#### Construction of the histogram

To construct the histogram of the distribution of Ln(PSA), we first determined the optimal size of the number of bins for each subset of the data. To determine the width of each bin, *h*, we used Freedman-Diaconis rule as follows^35^:

14 $$h = \frac{2\,IQR(x)}{{\sqrt[3]{n}}}$$

where *n* is the sample size, *x* is Ln(PSA), and *IQR*(*x*) is the interquartile range of *x*. The optimal number of bins, *n*_*bin*_, was then calculated as follows:

15 $$n_{bin} = \left\lceil {\frac{\max (x) - \min (x)}{h}} \right\rceil$$

The relative frequency of Ln(PSA) for each bin was then calculated.

### Decomposition of the frequency curve into its components

A nonlinear curve fitting function (*nlsLM()* from *minpack.ml* package for *R*) was used to figure out the optimal values of parameters of a binormal equation (Eq. 2) best fit to the frequency distribution (based on our hypothesis about the distribution of Ln(PSA) in non-diseased and diseased people)^36^. The function works based on the Levenberg–Marquardt nonlinear least-squares algorithm^37^. Constraints were imposed on the parameters *a, σ*_1_*,* and *σ*_2_ in Eq. 2—they could only assume non-negative values; *pr*, the prior probability (prevalence) of the disease, could only assume values in the close interval of [0, 1].

The *pr* for the disease for men aged ≥ 65 years was around 0.5. Under this circumstance, the two Gaussian-distributed components (the two terms in Eq. 2) have an almost equal contribution to shaping the total distribution of PSA (Fig. 1); thus, *μ*_2_ and *σ*_2_ would be calculated with the same accuracy as *μ*_1_ and *σ*_1_ are. As we hypothesized that the distribution of PSA in those with the disease is not age-dependent, we used the mean and SD of Ln(PSA) obtained for men aged ≥ 65 with the disease (*μ*_2_ and *σ*_2_) as an estimate for the distribution of diseased people for all other age groups studied. Therefore, in the first pass, we used a six-parameter binormal equation (Eq. 2) for curve fitting for the age group of ≥ 65 to find the best estimates for *μ*_2_ and *σ*_2_; then, we assumed these values fixed in the curve fitting looking for other parameters *a, pr, μ*_1_*,* and *σ*_1_, hence, a four-parameter model.

#### The reference range

In medical sciences, the reference range for diagnostic test results, here PSA, is important and commonly defined as the interval between the 2.5th and 97.5th percentiles of the test value distribution in an apparently healthy population^14^. Presuming a normal distribution for Ln(PSA) in non-diseased individuals and given the mean and SD of the distribution (*μ*_1_ and *σ*_1_) for each age group, the reference range was calculated as *μ*_1_ ± 1.96 *σ*_1_.

#### Prevalence of the disease

The prevalence of the disease for each age group was directly derived from the curve fitting procedure, the *pr* in Eq. 2.

#### Calculation of the sensitivity and specificity

Given the means and SDs of Ln(PSA) in diseased and non-diseased people and considering normal distribution for both diseased and non-diseased people (according to our hypothesis), we simply calculated the *Se* and *Sp* of a test assuming a cut-off value of *t* for Ln(PSA) using Eqs. 3 and 4, respectively^3^. Considering the normal distribution of the classifier in our case study, Ln(PSA), in both diseased and non-diseased people, given the cut-off value of *t*, we calculated the *Se* and *Sp* using the *R* function *pnorm()* as follows:

Se < − pnorm(t, mean = µ_2_, sd = σ_2_, lower.tail = FALSE)

Sp < − pnorm(t, mean = µ_1_, sd = σ_1_, lower.tail = TRUE)

#### The receiver operating characteristic curve and the cut-off value

The *Se* and *Sp* for a series of Ln(PSA) cut-off values were calculated, based on which a receiver operating characteristic (ROC) curve was constructed. Then, we calculated the most appropriate cut-off value for PSA^3^.

#### Positive and negative predictive values

Given the *Se*, *Sp*, and *pr*, we calculated the positive (*PPV*) and negative predictive values (*NPV*) using Eq. 5.

#### Calculation of the likelihood ratios

Given the *Se* and *Sp*, various types of likelihood ratios were calculated using Eqs. 6 to 8^8^. Considering the binormal distribution of Ln(PSA) in diseased and non-diseased men, we calculated the *LR* of *x* (Eq. 8), using the *R* function *dnorm()* that returns the density function of a normal distribution, as follows^8^:

LRx < − dnorm(x, mean = µ_2_, sd = σ_2_)/dnorm(x, mean = µ_1_, sd = σ_1_)

#### Ethics

The study was conducted in accordance with the Declaration of Helsinki Code of Ethics. The study protocol was approved by the Petroleum Industry Health Organization Institutional Review Board. Informed consents were obtained from all participants. The authors did not have access to identifiable data records.

## Supplementary information

Supplementary Information 1.

Supplementary Information 2.

## Data Availability

The raw data and codes written in *R* are available from the journal Web site.
