# Supplementary material for: On the information hidden in a classifier distribution
Source: Sci Rep. 2021 Jan 13;11:917. doi: 10.1038/s41598-020-79548-9 (PMC7807039; doi:10.1038/s41598-020-79548-9)
Supplement: Supplementary file 1 — Supplementary Information 1. [file 41598_2020_79548_MOESM1_ESM.docx]

**SUPPLEMENTARY DATA AND CODES**

**On the Information Hidden in a Classifier Distribution**

**Raw data:** The data are in a CSV file, *PSAcleanFiltered.csv*, attached.

***R* Codes used**

*## R Codes used for analyzing the data*

*## On the Information Hidden in a Classifier Distribution*

*library(minpack.lm)*

*#--- Normal function to be used by nonlinear curve fitting*

*f <- function(x, m, s)*

*{*

*return(dnorm(x, m, s))*

*}*

*#############################################################################################*

*# In the first pass, we used a six-parameter binormal equation (Eq 2 of the article) for curve
# fitting for the age group of ≥65 to find the best estimates for μ2 and σ2 (m2 and s2 in the
# program); then, we assumed these values fixed in the curve fitting looking for other parameters
# a, pr, μ1 (m1 in the program), and σ1 (s1 in the program), hence, a four-parameter model.*

*#*

*# The lines of codes used in the first pass are thus commented.*

*#*

*##=================================================================*

*##--- Reading the data from the file after data cleaning*

*## only data of men aged 20 yrs or more and 0.1 < PSA < 100 ng/mL*

*#*

*# data<-read.csv("PSAcleanFiltered.csv", header=TRUE)*

*#############################################################################################*

*##======= DISCOVERING THE MEAN AND SD OF CANCEROUS PATIENTS =======*

*##--- To determine m2 and s2*

*# Filter <- data$Age>=65*

*#*

*# Age <- data$Age[Filter]*

*# PSA <- data$PSA[Filter]*

*# LnPSA <- log(PSA)*

*#*

*# nsample <- length(PSA)*

*#*

*#------- Freedman Diaconis's Rule for bin size --------------*

*# h <- 2 * IQR(LnPSA) / nsample^(1/3)*

*# n <- ceiling((max(LnPSA) - min(LnPSA))/h)*

*# br <- seq(min(LnPSA), max(LnPSA), length = n)*

*#*

*# x <- seq((br[1]+br[2])/2, by = h, length = n-1)*

*#*

*#--- Determining the relative frequency distribution of LnPSA*

*# freqLnPSA <- table(cut(LnPSA, breaks = br))*

*# names(freqLnPSA) <- x*

*# freqLnPSA <- as.data.frame(freqLnPSA)*

*# names(freqLnPSA) <-c ("x", "y")*

*# freqLnPSA$x <- as.numeric(levels(freqLnPSA$x))*

*# freqLnPSA$y <- freqLnPSA$y/sum(freqLnPSA$y)*100 # Relative freq (%)*

*#*

*##--- Nonlinear regression*

*# st <- list(a=10, pr=0.5, m1=-0.2, s1=0.7, m2=1.0, s2=0.8) # First guess for parameters*

*# nl <- nlsLM(freqLnPSA$y ~ a*(1-pr)*f(freqLnPSA$x, m1, s1)+a*pr*f(freqLnPSA$x, m2, s2),*

*# data=freqLnPSA, start=st, lower=c(0, 0, -Inf, 0, -Inf, 0),*

*# upper=c(Inf, 1, Inf, Inf, Inf, Inf))*

*#*

*# m2 <- as.numeric(coef(nl)[5])*

*# s2 <- as.numeric(coef(nl)[6])*

*#*

*#####################################################################################*

*#=============================== ANALYSIS OF DATA SUBSET =======================*

*# derived from phase 1 (see comments above)*

*m2 <- 1.033031*

*s2 <- 0.7664218*

*#--- Reading the data from the file after data cleaning*

*# only data of men aged 20 yrs or more and 0.1 < PSA < 100 ng/mL*

*data<-read.csv("PSAcleanFiltered.csv", header = TRUE)*

*#======= DISCOVERING THE MEAN AND SD OF NORMAL PEOPLE ==========================*

*Filter <- data$Age >= 54 & data$Age < 60 # Defining a filter for age subset*

*Age <- data$Age[Filter]*

*PSA <- data$PSA[Filter]*

*LnPSA <- log(PSA)*

*nsample <- length(PSA)*

*#------- Freedman Diaconis's Rule for bin size --------------------------------------*

*h <- 2 * IQR(LnPSA) / nsample^(1/3)*

*n <- 1 + as.integer((max(LnPSA) - min(LnPSA)) / h)*

*br <- seq(min(LnPSA), max(LnPSA), length = n)*

*x <- seq((br[1]+br[2])/2, by = h, length = n-1)*

*#--- Determining the relative frequency distribution of LnPSA*

*freqLnPSA <- table(cut(LnPSA, breaks=br))*

*names(freqLnPSA) <- x*

*freqLnPSA <- as.data.frame(freqLnPSA)*

*names(freqLnPSA) <- c("x", "y")*

*freqLnPSA$x <- as.numeric(levels(freqLnPSA$x))*

*freqLnPSA$y <- freqLnPSA$y/sum(freqLnPSA$y) * 100 # Relative freq (%)*

*#--- Nonlinear 4-parameter curve fitting, knowing the m2 and s2*

*st <- list(a=10, pr=0.5, m1=-0.2, s1=0.7) # First guess for parameters*

*nl <- nlsLM(freqLnPSA$y ~ a*(1-pr)*f(freqLnPSA$x, m1, s1)+a*pr*f(freqLnPSA$x, m2, s2),*

*data=freqLnPSA, start=st, lower=c(0, 0, -Inf, 0), upper=c(Inf, 1, Inf, Inf))*

*a <- as.numeric(coef(nl)[1])*

*pr <- as.numeric(coef(nl)[2])*

*m1 <- as.numeric(coef(nl)[3])*

*s1 <- as.numeric(coef(nl)[4])*

*# Output the distribution graph*

*fname <- paste0("Fig ", range(Age)[1], "-", range(Age)[2])*

*jpeg(file = paste(fname, ".jpg"), width = 2400, height = 2000, units = "px")*

*par(cex=7, mai=c(6, 6, 3, 3))*

*plot(freqLnPSA$x, freqLnPSA$y, col="red", type="n", bty="n", ann = FALSE)*

*mtext(side = 1, text = "Ln(PSA)", line = 2.5, cex=7)*

*mtext(side = 2, text = "Relative Frequncy (%)", line = 2.5, cex=7)*

*yn <- a * (1-pr) * f(LnPSA, m1, s1) # for diseased people*

*yd <- a * pr * f(LnPSA, m2, s2) # for non-diseased people*

*# drawing the histogram*

*segments(br[1], 0, br[1], freqLnPSA$y[1])*

*for (i in 1:(n-1))*

*{*

*segments(br[i], freqLnPSA$y[i], br[i+1], freqLnPSA$y[i])*

*segments(br[i+1], freqLnPSA$y[i], br[i+1], freqLnPSA$y[i+1])*

*}*

*segments(br[n], freqLnPSA$y[n], br[n], 0)*

*segments(br[1], 0, br[n], 0)*

*# creating transparent colors*

*transorange = rgb(255, 165, 0, max=255, alpha = 130)*

*transblue = rgb(0, 0, 255, max=255, alpha = 170)*

*transmagenta = rgb(255, 0, 255, max=255, alpha = 150)*

*lines(LnPSA, yn+yd, col=transorange, lwd=10, ylab="") # the fitted binormal curve*

*lines(LnPSA, yn, col=transblue, lwd=5, lty=2, ylab="") # the normal curve for non-diseased*

*lines(LnPSA, yd, col=transmagenta, lwd=5, lty=2, ylab="") # the normal curve for diseased*

*abline(v=m1-1.96*s1, col="forestgreen", lwd=3) # lower limit of the reference range*

*abline(v=m1+1.96*s1, col="forestgreen", lwd=3) # upper limit of the reference range*

*dev.off()*

*RSS <- sum(residuals(nl)^2)*

*TSS <- sum((freqLnPSA$y - mean(freqLnPSA$y))^2)*

*R.square <- 1 - (RSS/TSS)*

*##############################################################*

*#*

*#------- Calculation of cut-off values, Se, Sp, PPV, NPV and LRs*

*#C <- 4 # presumed cost of false-negative relative to false-positive results;*

*# for more information see doi: 10.11613/BM.2016.034*

*C <- (1-pr)/pr # Max Youden's index*

*d <- (m2 - m1)/s1*

*s <- s2/s1*

*cut <- (s*sqrt(2*(s^2-1)*log(s*(1-pr)/(C*pr))+d^2)-d)/(s^2-1)*

*cut <- m1 + cut*s1*

*Se <- pnorm(cut, m2, s2, lower.tail=FALSE)*

*Sp <- pnorm(cut, m1, s1, lower.tail=TRUE)*

*PPV <- (Se*pr)/(Se*pr+(1-Sp)*(1-pr))*

*NPV <- Sp*(1-pr)/(Sp*(1-pr)+(1-Se)*pr)*

*NNM <- 1/(pr * (1-Se) + (1-pr)*(1-Sp))*

*LRpos <- Se/(1-Sp)*

*LRneg <- (1-Se)/Sp*

*LR <- dnorm(freqLnPSA$x, m2, s2)/dnorm(freqLnPSA$x, m1, s1)*

*outfp <- file("Out.txt", open="a")*

*writeLines("\n----------------------------", outfp)*

*writeLines(paste0("Age Range: ", min(Age),"-", max(Age), "\tn: ", nsample), outfp)*

*writeLines(paste0("Reference Range; 95th %ile: ", round(exp(m1-1.96*s1), digits=2), "-",*

*round(exp(m1+1.96*s1), digits=2), ";\t", round(exp(m1+1.65*s1), 2)), outfp)*

*writeLines(paste0("Prevalence: ", round(pr, digits=3)), outfp)*

*writeLines(paste0("Cut-off value: ", round(exp(cut), digits=2)), outfp)*

*writeLines(paste0("Sensitivity: ", round(Se, digits=3), "\tSpecificity: ", round(Sp, digits=3)), outfp)*

*writeLines(paste0("PPV: ", round(PPV, digits=3), "\tNPV: ", round(NPV, digits=3)), outfp)*

*writeLines(paste0("NNM: ", round(NNM, digits=1)), outfp)*

*writeLines(paste0("LR+ : ", round(LRpos, digits=2), "\tLR- :", round(LRneg, digits=2)), outfp)*

*writeLines("\nPSA\tLR", outfp)*

*writeLines(paste0(round(exp(freqLnPSA$x), digits=2), "\t", round(LR, digits=2)), outfp)*

*#======= Constructing the ROC*

*Se <- pnorm(freqLnPSA$x, m2, s2, lower.tail=FALSE)*

*Sp <- pnorm(freqLnPSA$x, m1, s1, lower.tail=TRUE)*

*auc <- 0 # area under the curve*

*for (i in 1:(length(Se)-1)) # using trapezoid*

*{*

*auc<-auc+(Sp[i+1]-Sp[i])*(Se[i]+Se[i+1])/2*

*}*

*#======== Drawing the ROC*

*fname <- paste0("ROC ", range(Age)[1], "-", range(Age)[2])*

*jpeg(file = paste(fname, ".jpg"), width = 2000, height = 2000, units = "px")*

*par(cex=3.2, mai=c(4.0, 6, 3, 3))*

*plot(1-Sp, Se, col="black", type="n", bty="n", ann = FALSE, xlim=c(0, 1), ylim=c(0, 1), asp=1)*

*lines(1-Sp, Se, col="magenta", lwd=8)*

*lines(c(0, 1), c(0, 1), col="black", lty=2, lwd=3)*

*Se <- pnorm(cut, m2, s2, lower.tail=FALSE)*

*Sp <- pnorm(cut, m1, s1, lower.tail=TRUE)*

*points(1-Sp, Se, pch=16, col="blue", cex=2) # draw the cut-off point*

*mtext(side = 1, text = "1 - Sp", line = 2.5, cex=4)*

*mtext(side = 2, text = "Se", line = 2.5, cex=4)*

*writeLines(paste0("\nR^2: ", round(R.square, digits=3), "\tAUC:", round(auc, digits=3)), outfp)*

*close(outfp)*

*dev.off()*
